# The Value of the Reflex Arc in Diagnosis

**Published:** 1906-10

**Authors:** Marc Ray Hughes

**Affiliations:** Professor of Mental and Nervous Diseases, Barnes University, St. Louis


For Texas Medical Journal.

### The Value of the Reflex Arc in Diagnosis.

BY MARC RAY HUGHES, M. D.,
Professor of Mental and Nervous Diseases, Barnes University, St. Louis.

In the different cord lesions that the neurologist has to deal with it is obvious that the value of a thorough conception as to the value and understanding of the reflex arcs and the light that they may shed upon an obscure condition is imperative. There are times, of course, in which there is a minus reflex action of some of the parts without a gross pathological lesion; and again, on the other hand, there might be an exaggerated condition; however, that condition to which I refer as being capable of producing both an exaggerated and a diminished reflex is comparatively rare; that is, the profoundness of the disease, namely, hysteria. It, in itself, is common enough, though its resultant, a minus or a highly plus reflex, is rare. Especially is that true of an absent reflex.

When the reflex is diminished and not entirely lost, as we sometimes see it in the profound types of hysteria, there is that character to it that enables us to distinguish between a diminution of reflex action, hysterical conditions, and a diminution from breaking down of tissue or some gross structural change in the cord.

It is by no means a hard and fast rule that all absence of reflex movements below certain planes means a disintegration of tissue within the cord, or even an exudate that might produce a diminished or even an absent reflex action.

I am inclined to believe that we lay too much stress upon a diminished or an exaggerated reflex movement, as is clearly shown in cases where reinforcement changes the whole situation. Where we have clear conception as to the action of motor and sensory nerve communication from the brain and spinal cord to their periphery, and understand the effect of phenomena of mental forces, and how the centrifugal and centripetal powers retard or accelerate, or apparently (and sometimes really do) stimulate these nerves through its action on the vascular system, we can but stop and think that while the absence or acceleration of reflex movement is by all means an important factor in diagnosis, at the same time it is just as important to consider the mental environment of the patient, the effect of suggestion, and other conditions bearing upon the individual's mental poise, in which the two governing powers may throw their influence and as a resultant leave either a minus or a plus reflex in an inorganic disease.

